# Supplementary figures and images for: Analysis of opticin binding to collagen fibrils identifies a single binding site in the gap region and a high specificity towards thin heterotypic fibrils containing collagens II, and XI or V/XI
Source: PLoS One. 2020 Aug 7;15(8):e0234672. doi: 10.1371/journal.pone.0234672 (PMC7413481; doi:10.1371/journal.pone.0234672)

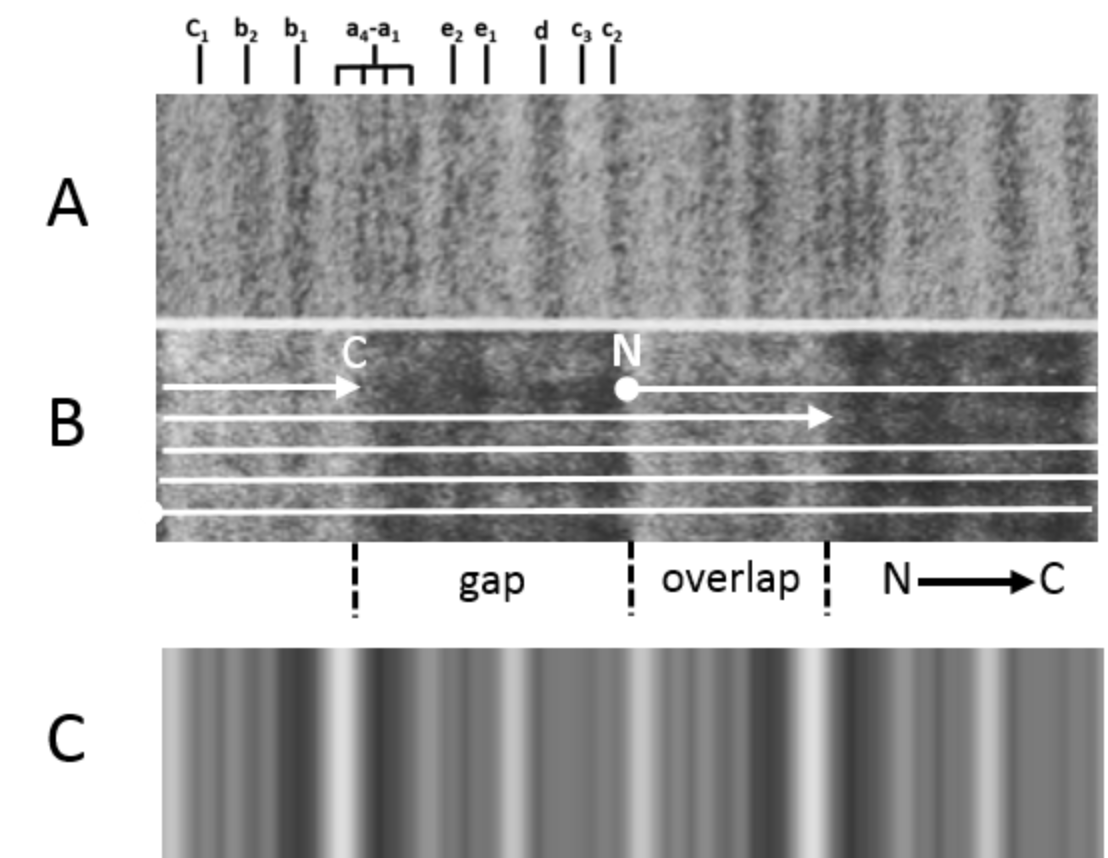

Supplement: S1 Fig — (A) and (B) show the positive and negative stain patterns, respectively, of collagen I fibrils. The 12 stain lines of the positive stain pattern (A) are labelled with the standard nomenclature [15]. The gap-overlap structure is indicated on the negative stain pattern [20] along with the axial locations of the N- and C-ends of the collagen molecules (B); the molecular polarity is indicated by the arrow. (C) shows the average negative stain pattern for the vitreous fibrils in the present study oriented and aligned with the collagen I stain patterns. (TIF) [file pone.0234672.s001.tif]

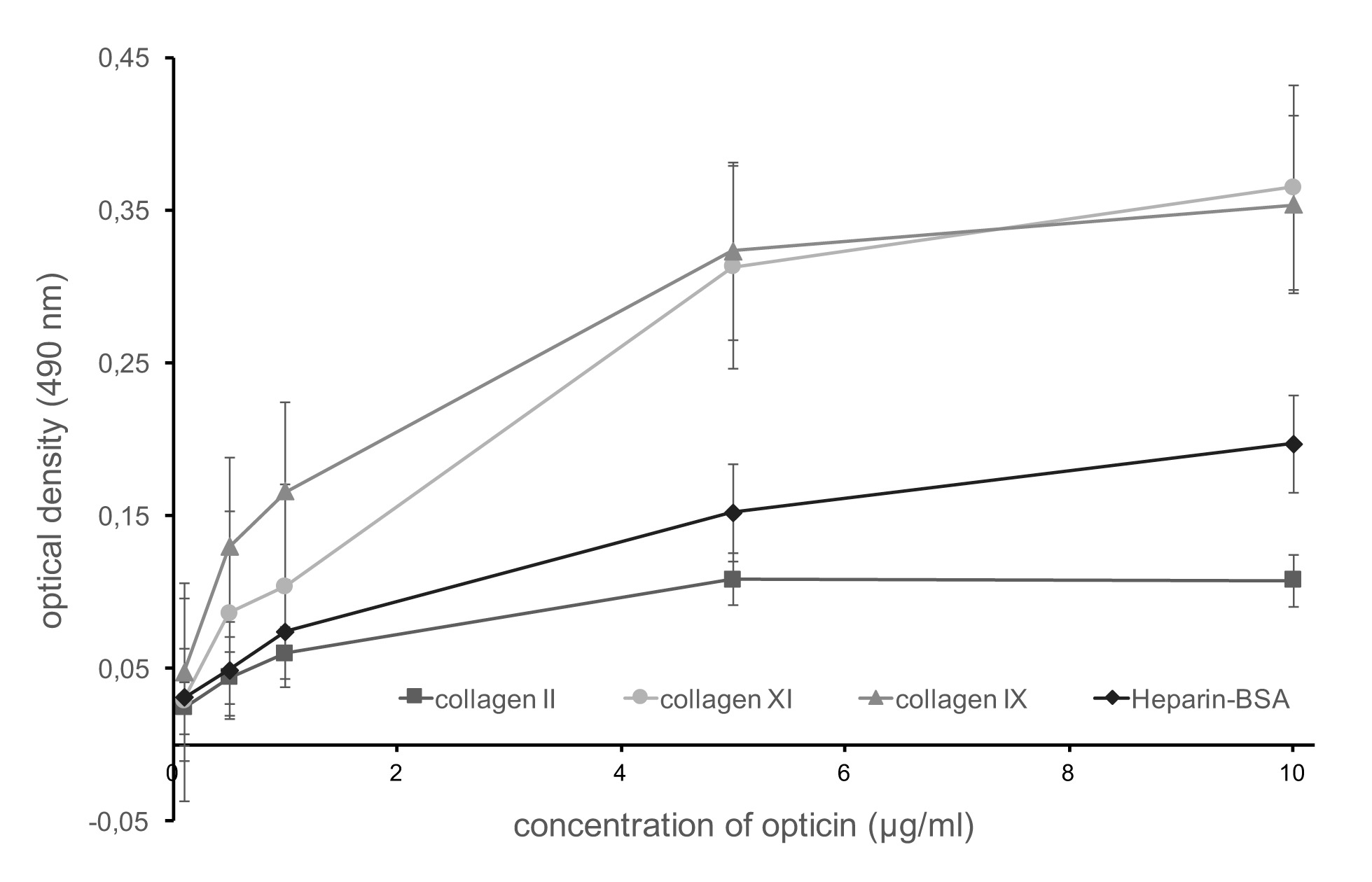

Supplement: S2 Fig — Opticin showed concentration-dependent binding to collagens II, IX and XI (heparin albumin was used as a positive control). (TIF) [file pone.0234672.s002.tif]

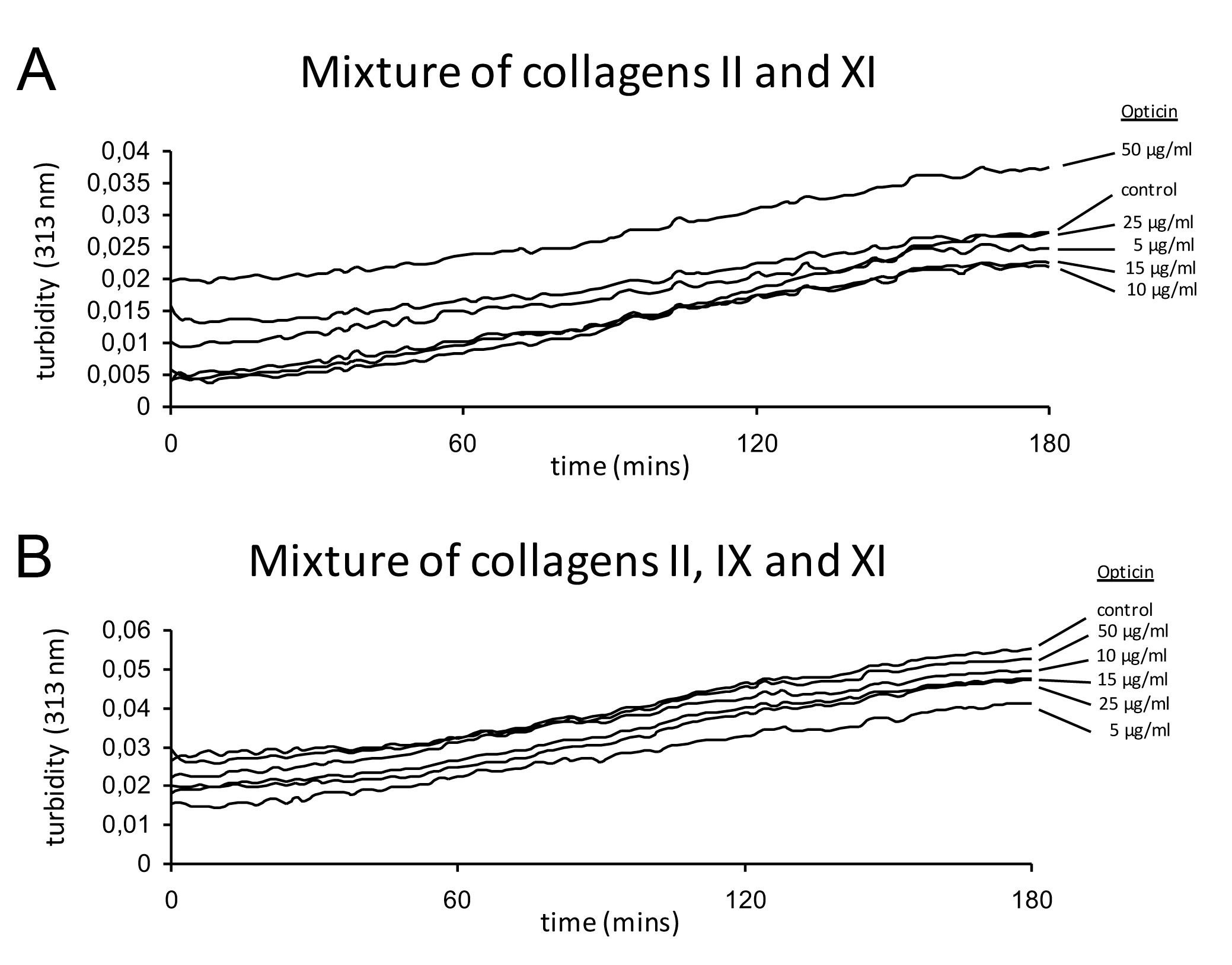

Supplement: S3 Fig — Turbidity measurements at 313 nm during fibrillogenesis with collagens II and XI or collagens II, IX and XI in the presence of varying concentrations of opticin. (TIF) [file pone.0234672.s003.tif]

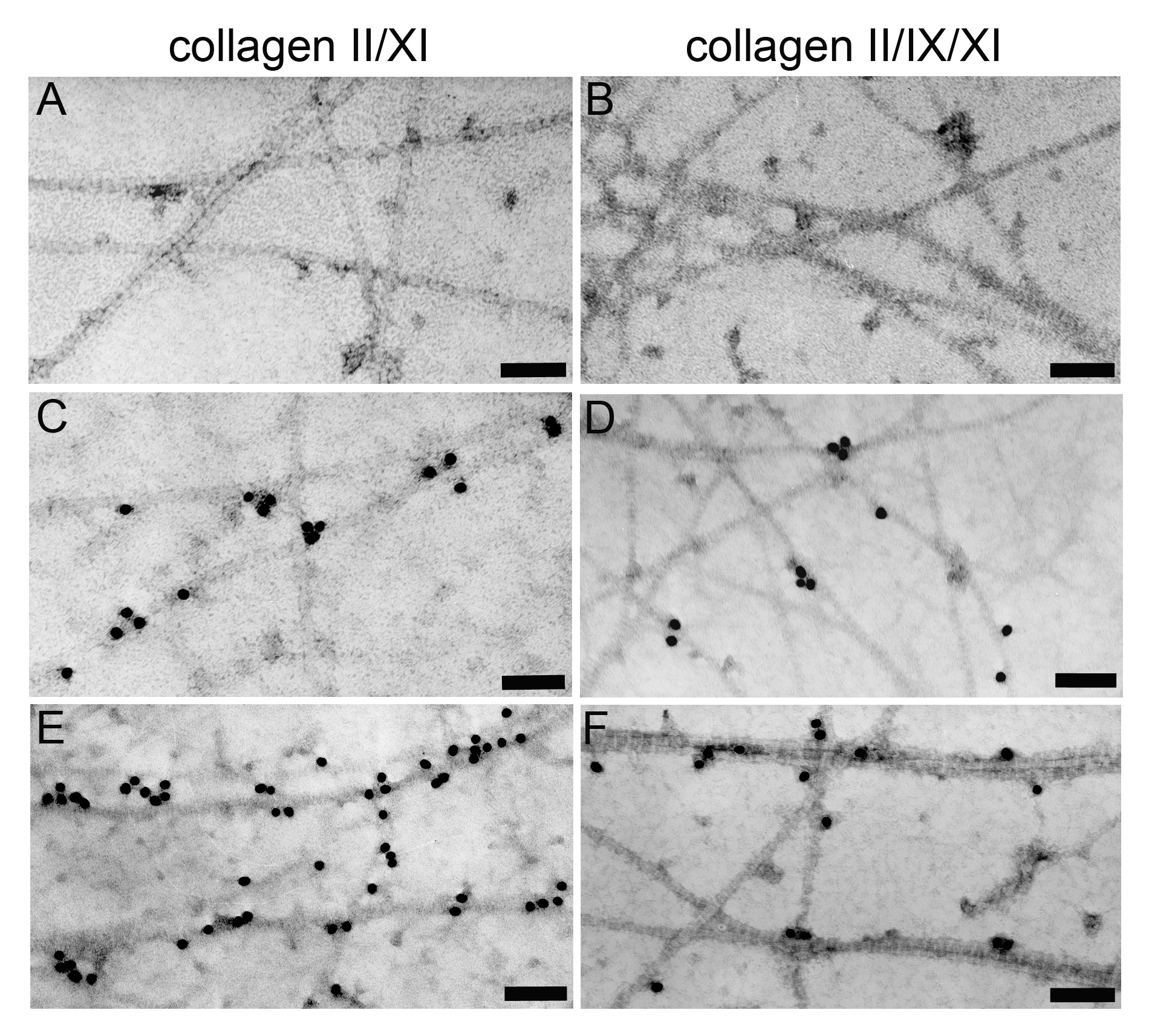

Supplement: S4 Fig — Collagen fibrils reconstituted from mixtures of collagens II and XI (A,C,E) and collagens II, IX, and XI (B,D,F) followed by immunoelectron microscopy with opticin antibody and gold conjugated secondary antibody. There was no labelling in the absence of opticin (A,B); when the fibrils were reconstituted in the presence of 5 μg/ml of opticin, immunogold labelling was observed (C,D), and increased labelling was observed when reconstituted with 25 μg/ml of opticin (E,F), (bars 100 nm). (TIF) [file pone.0234672.s004.tif]

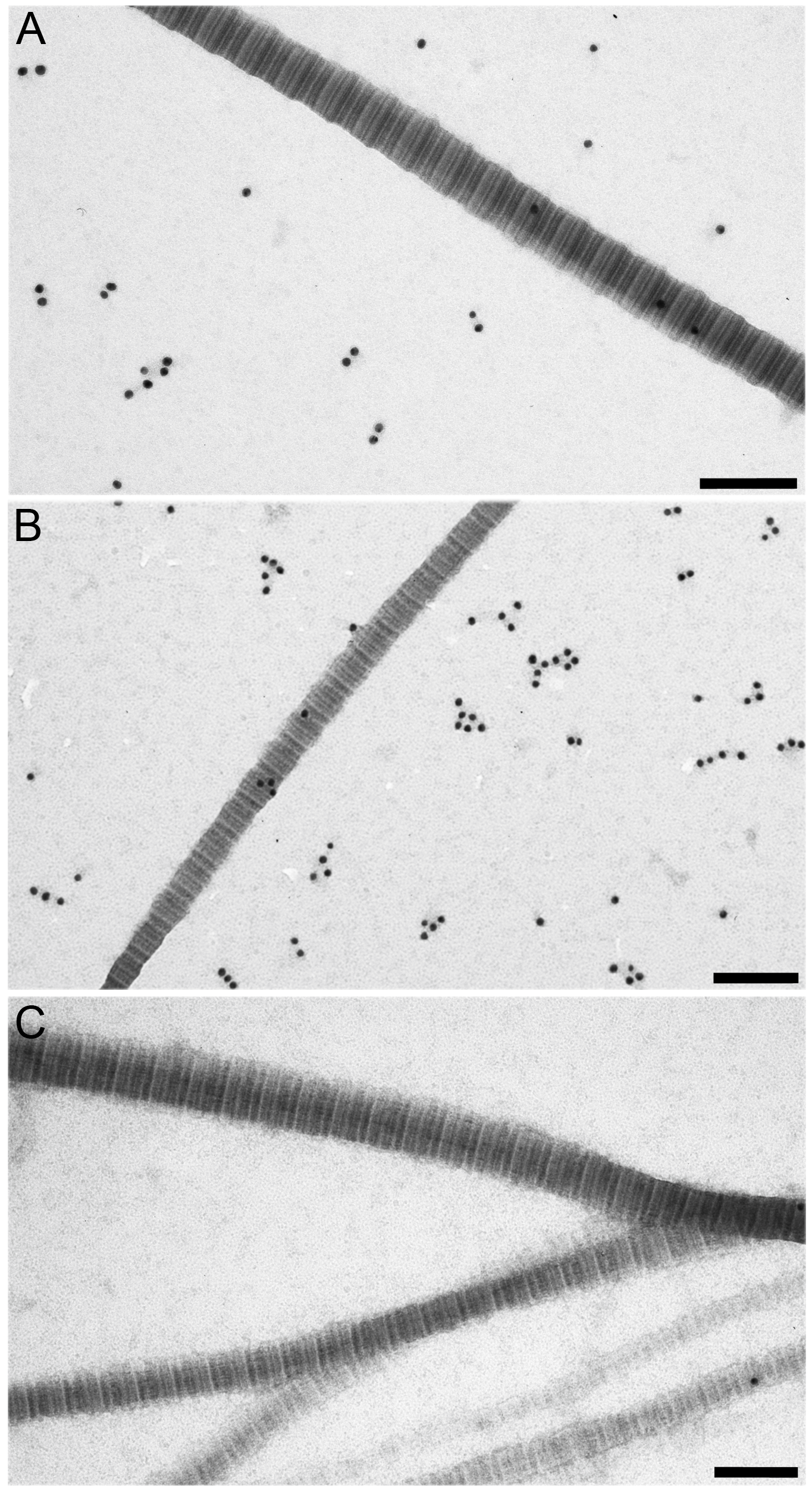

Supplement: S5 Fig — Collagen I was purified in a native and fibrillogenesis-competent form from tarso-metatarsal tendons of 17-day-old chicken embryos as described previously [1]. Fibrils were formed in vitro then immunoelectron microscopy using opticin antibodies and gold-labelled secondary antibody was performed as described in Materials and Methods. Fibrils were incubated with 5 μg/ml of opticin (A), 50 μg/ml of opticin (B) or in control experiments an equal volume of storage buffer without opticin (C), (bars 200 nm). (TIF) [file pone.0234672.s005.tif]
