# Supplementary material for: Analysis of opticin binding to collagen fibrils identifies a single binding site in the gap region and a high specificity towards thin heterotypic fibrils containing collagens II, and XI or V/XI
Source: PLoS One. 2020 Aug 7;15(8):e0234672. doi: 10.1371/journal.pone.0234672 (PMC7413481; doi:10.1371/journal.pone.0234672)
Supplement: S1 Text — (DOCX) [file pone.0234672.s006.docx]

S5. Reference

1. Hansen U, Bruckner P. Macromolecular specificity of collagen fibrillogenesis: fibrils of collagens I and XI contain a heterotypic alloyed core and a collagen I sheath. J Biol Chem. 2003; 278: 37352-37359.
